# Low Virulence of the Fungi *Escovopsis* and *Escovopsioides* to a Leaf-Cutting Ant-Fungus Symbiosis

**DOI:** 10.3389/fmicb.2021.673445

**Published:** 2021-07-29

**Authors:** Débora Mello Furtado de Mendonça, Marcela Cristina Silva Caixeta, Gabriel Leite Martins, Camila Costa Moreira, Thiago Gechel Kloss, Simon Luke Elliot

**Affiliations:** ^1^Department of Entomology, Federal University of Viçosa, Viçosa, Brazil; ^2^Department of Entomology and Acarology, Luiz de Queiroz College of Agriculture, University of São Paulo, Piracicaba, Brazil; ^3^Department of Biological Sciences, Minas Gerais State University, Ubá, Brazil

**Keywords:** host-parasite interactions, parasitism, Atta, Acromyrmex, evolution of virulence, Hypocreales, Formicidae

## Abstract

Eusocial insects interact with a diversity of parasites that can threaten their survival and reproduction. The amount of harm these parasites cause to their hosts (i.e., their virulence) can be influenced by numerous factors, such as the ecological context in which the parasite and its host are inserted. Leaf-cutting ants (genera *Atta*, *Acromyrmex* and *Amoimyrmex*, Attini: Formicidae) are an example of a eusocial insect whose colonies are constantly threatened by parasites. The fungi *Escovopsis* and *Escovopsioides* (Ascomycota: Hypocreales) are considered a highly virulent parasite and an antagonist, respectively, to the leaf-cutting ants’ fungal cultivar, *Leucoagaricus gongylophorus* (Basidiomycota: Agaricales). Since *Escovopsis* and *Escovopsioides* are common inhabitants of healthy colonies that can live for years, we expect them to have low levels of virulence. However, this virulence could vary depending on ecological context. We therefore tested two hypotheses: (i) *Escovopsis* and *Escovopsioides* are of low virulence to colonies; (ii) virulence increases as colony complexity decreases. For this, we used three levels of complexity: queenright colonies (fungus garden with queen and workers), queenless colonies (fungus garden and workers, without queen) and fungus gardens (without any ants). Each was inoculated with extremely high concentrations of conidia of *Escovopsis moelleri*, *Escovopsioides nivea*, the mycoparasitic fungus *Trichoderma longibrachiatum* or a blank control. We found that these fungi were of low virulence to queenright colonies. The survival of queenless colonies was decreased by *E. moelleri* and fungus gardens were suppressed by all treatments. Moreover, *E. nivea* and *T. longibrachiatum* seemed to be less aggressive than *E. moelleri*, observed both *in vivo* and *in vitro*. The results highlight the importance of each element (queen, workers and fungus garden) in the leaf-cutting ant-fungus symbiosis. Most importantly, we showed that *Escovopsis* may not be virulent to healthy colonies, despite commonly being described as such, with the reported virulence of *Escovopsis* being due to poor colony conditions in the field or in laboratory experiments.

## Introduction

Parasites can play an important role in many aspects of their hosts’ life, threatening their survival and reproduction. The harm that parasites cause to their hosts, referred to as virulence (Frank, 1996), can be influenced by numerous factors, involving traits related to the parasite and to its host, in addition to the environmental conditions in which both are to be found. Theory about virulence evolution predicts that there is a relationship between parasites’ virulence and their mode of transmission to new hosts (Ewald, 1987; Alizon et al., 2009). It has been suggested that vertically transmitted parasites tend to be less virulent in relation to horizontally transmitted parasites, because their fitness depends on their host’s reproductive success (Clayton and Tompkins, 1994). This has been demonstrated in some empirical studies (Bull et al., 1991; Clayton and Tompkins, 1994; Tompkins et al., 1996; Agnew and Koella, 1997; Stewart et al., 2005; Pagán et al., 2014) although it cannot be taken as a general rule, especially as many parasites can present both modes of transmission (Ebert, 2013; Cressler et al., 2015). Host lifespan can also be an important factor in understanding the evolution of a parasite (Watson, 2013). It is predicted that hosts with a shorter lifespan reduce the future opportunities of parasite transmission, thus parasites should grow faster within the host and be transmitted earlier, which is reflected in a higher virulence (Nidelet et al., 2009). In addition, the virulence of some parasites may depend plastically on the immediate context in which they are inserted; they may for example increase their virulence when their hosts are stressed (Brown et al., 2000, 2003; Jokela et al., 2005; Manley et al., 2017).

As with all living organisms, eusocial insects such as ants, termites and some bees and wasps may be exposed to a diversity of parasites. These parasites include viruses, bacteria and fungi, among others (Schmid-Hempel, 1998) and the manner in which sociality could affect their evolution has been a topic of some discussion. Some authors argue that long-lived colonies of insect societies represent a buffered and homeostatic environment, which tends to lead to a reduction in parasite virulence (e.g., Hughes et al., 2008). Additionally, highly genetically diverse colonies of some eusocial insects, that have polyandrous queens (i.e., multiple mating queen with several males), have been associated with a lower incidence of disease and better resistance to parasite infection (Hughes and Boomsma, 2004; Tarpy and Seeley, 2006; Seeley and Tarpy, 2007). Furthermore, the level of a parasite’s virulence can increase in some stressful stages of a eusocial insects’ life history, such as the colony-founding period (Brown et al., 2003).

Among eusocial insects, the leaf-cutting ants (genera *Atta*, *Acromyrmex* and *Amoimyrmex*, Attini: Formicidae) are well-known to interact with a diversity of microorganisms that are present in their colonies, including parasites and mutualists (Pagnocca et al., 2012). These ants cultivate the fungus *Leucoagaricus gongylophorus* (Basidiomycota: Leucocoprineae, Agaricaceae), using it as food, while providing nutrients, protection and dispersion in return. The fungal cultivar of leaf-cutting ants is farmed in subterraneous chambers that shelter the “fungus garden.” This structure is composed of the fungal cultivar mycelium and fragments of leaves and flowers that serve as a substrate to the fungus’ growth. It is important to note that fungus gardens also harbor a range of bacteria, yeasts and filamentous fungi (Carreiro et al., 1997; Currie et al., 1999a; Rodrigues et al., 2005b, 2008; Suen et al., 2010).

Fungi of the genera *Escovopsioides* and *Escovopsis* (Ascomycota: Hypocreales) are examples of microorganisms that are commonly found in the fungus gardens (Augustin et al., 2013; Reis et al., 2015). The genus *Escovopsioides* includes only one species described to date, *Escovopsioides nivea* (Augustin et al., 2013) and its role in colonies of leaf-cutting ants is as yet poorly understood. A study performed by Varanda-Haifig et al. (2017) demonstrated that this fungus is an antagonist of *L. gongylophorus* capable of inhibiting the growth of the fungal cultivar in culture medium. Negative effects of *E. nivea*, such as degradation of infected parts leading to removal by ants, have also been demonstrated in small fungus garden fragments; this is in addition to inhibition of growth of *L. gongylophorus* in dual-culture bioassays with *E. nivea* (Osti and Rodrigues, 2018). The genus *Escovopsis* is phylogenetically related to *Escovopsioides* (Augustin et al., 2013) and is considered a specialized parasite of the fungal cultivar of leaf-cutting ants (Currie et al., 1999a). Some early studies suggested that this parasite is highly virulent to its host, capable of causing the death of infected colonies and reducing the fungus garden biomass as well as the production of new ant individuals (Currie et al., 1999a; Currie, 2001). Based on the results of these studies, it has become disseminated in the literature that this fungus actually represents a highly virulent parasite (Currie et al., 1999a, 2003a,b; Currie, 2001; Currie and Stuart, 2001; Stearns and Hoekstra, 2005; Hölldobler and Wilson, 2009; Farji-Brener et al., 2016; Verza et al., 2017). However, it is important to consider some points related to this. Firstly, *Escovopsis* is often found in the fungus garden of healthy colonies that remain foraging and growing (Currie et al., 1999a; Gerardo et al., 2004; Rodrigues et al., 2005a; Augustin, 2011). Secondly, one of the pioneering studies that investigated the impact of *Escovopsis* on the ant-fungus symbiosis was conducted using newly founded colonies of 10–12 weeks (Currie, 2001). Thus, it is possible these colonies were still fragile and, in this condition, could suffer a heightened negative impact because of this parasite. Thirdly, some studies considering the mode of *Escovopsis* transmission between colonies of leaf-cutting ants have implied that the parasite may to be horizontally transmitted (Currie et al., 1999a; Moreira et al., 2015; Augustin et al., 2017) which has been taken spuriously as evidence of high virulence (as vertical transmission selects for lower virulence – see above) (Currie et al., 1999a). Nevertheless, the results that indicated horizontal transmission of *Escovopsis* do not exclude the possibility of vertical transmission, which could support a low virulence consistent with theory of virulence evolution. Fourth, horizontal transmission does not automatically indicate high virulence as in the case of the common cold of humans for example. Finally, another important issue is that, like other parasites, the level of virulence presented by *Escovopsis* may also vary depending on the ecological context in which it is inserted, for example, when the host is weakened or experiencing stressful conditions.

It is also important to consider that colonies of leaf-cutting ants function as superorganisms. Besides the fungus gardens, the reproductive and non-reproductive castes are involved in different functions, such as reproduction, defence and foraging. These individuals act similarly to the germ and somatic cells in the body of multicellular organisms (Cremer and Sixt, 2009). Thus, all elements that comprise the colonies act as a cooperative unit (Detrain and Deneubourg, 2006) and its components cannot survive and reproduce without one another (Cremer et al., 2017). In this sense, studies that investigate the impact of *Escovopsis* and *Escovopsioides* to the fungal cultivar of leaf-cutting ants must be conducted considering the role of the ants in the symbiosis as well as the other elements that comprise the colonies. Nevertheless, some studies that evaluated the impact of *Escovopsis* have been performed using *in vitro* assays (Folgarait et al., 2011a, b; Marfetán et al., 2015; Silva et al., 2006; Varanda-Haifig et al., 2017), colonies without queens (referred to as queenless colonies) or the fungus gardens without ants (Wallace et al., 2014). These studies are very important to understand the antagonism of *Escovopsis* and *Escovopsioides* with *L. gongylophorus* and how this may affect leaf-cutting ant colonies. However, since the virulence of parasites can be influenced by several factors, such as transmission mode of parasite and host lifespan, it may be that the use of only parts of the superorganism to conduct experiments also influences the level of virulence presented by these antagonistic fungi. In this context, our objective was to test two hypotheses: first, *Escovopsis* and *Escovopsioides* are of low virulence to whole colonies of leaf-cutting ants; second, the virulence of these fungi varies according to the level of complexity that a colony presents.

Here, we considered that a parasite with low virulence can cause some negative impact to its host, yet not enough to compromise its survival. On the other hand, a highly virulent parasite is able to cause its host’s death. In addition, we also considered that a higher level of colony complexity involves the interaction between the queen and workers as well as the fungus gardens (which include the fungal cultivar and the other microorganisms that are also present). When we disregard one or more components of this symbiosis, the level of complexity of this interaction decreases. The fungal cultivar *L. gongylophorus* cultivated in culture medium (*in vitro*) was considered the simplest level of the interaction since it excludes all the other elements that compromise a colony. Therefore, we considered that colonies, queenless colonies, fungus gardens and only *L. gongylophorus in vitro* represent decreasing levels of complexity. Our expectation was that *Escovopsis* and *Escovopsioides* would not represent a threat to a higher level of complexity, however, the virulence of these fungi would increase as the complexity levels of the colonies decrease.

## Materials and Methods

### Experimental Approach

**FIGURE 1:**
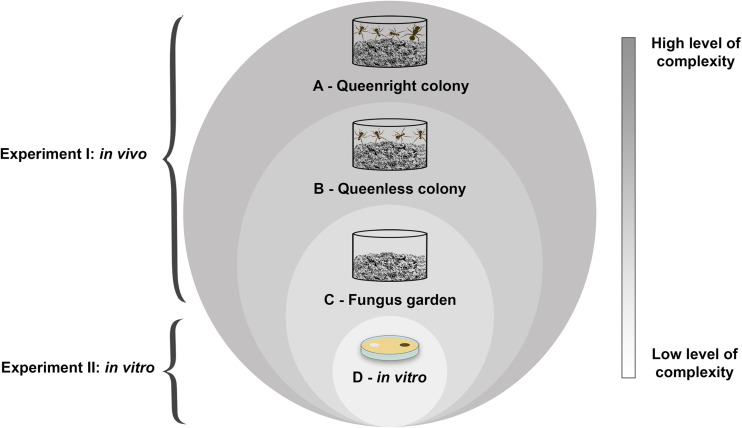
Schematic representation of experiments I and II showing the complexity levels of leaf-cutting ant-fungus symbiosis: **(A)** queenright colony; **(B)** queenless colony; **(C)** fungus garden; **(D)** fungal cultivar (*in vitro*). Note that a queenright colony is composed of the fungus garden, the queen and specialized workers with different functions. Therefore, we considered in our study that a queenright colony encompasses all these elements. On the other hand, a queenless colony does not contain an essential component of the queenright colony, the queen. As we disregard one or more organisms involved in the association between leaf-cutting ants and its fungal cultivar, the complexity levels of this interaction decrease. This can also be observed in fungus gardens without ants as well as the cultivation of the fungal cultivar *in vitro*, which we considered the simplest level of this interaction. From these considerations, we evaluated the impact of the fungi *Escovopsis* and *Escovopsioides* on *Acromyrmex subterraneus subterraneus* colonies and their fungal cultivar in different complexity levels.

We conducted two experiments to investigate the impact of *Escovopsis* and *Escovopsioides* on the leaf-cutting ant-fungus symbiosis. In the first experiment (Experiment I), we evaluated the impact of these fungi, *in vivo*, on leaf-cutting ant colonies and in three different levels of complexity: (i) queenright colonies (fungus garden + queen + workers; Figure 1A), (ii) queenless colonies (fungus garden + workers, without queen; Figure 1B) and (iii) fungus gardens (fungus gardens without any ants; Figure 1C). For this, we exposed the different levels of colonies to the following treatments: (a) conidial suspensions of *Escovopsis moelleri*, (b) *Escovopsioides nivea*, (c) *Trichoderma longibrachiatum*, and (d) blank control (0.01% Tween 80^®^ + NaCl 0.85% solution). In the second experiment (Experiment II), we evaluated the interaction of *E moelleri*, *E. nivea*, and *T. longibrachiatum* with the fungal cultivar *in vitro* through a paired culture bioassay (Figure 1D). The purpose of this experiment was to exclude most of the elements that comprise a colony, resulting in the simplest level of complexity, composed of only the fungal cultivar, *L. gongylophorus*. Thus, from these two experiments, we tested the first hypothesis, that *Escovopsis* and *Escovopsioides* are of low virulence for leaf-cutting ants colonies and the second, that this virulence increases as colony complexity decreases.

### Organisms

Twelve colonies of *Acromyrmex subterraneus subterraneus* (∼1-year-old) were collected in three areas on the Campus of Universidade Federal de Viçosa (UFV), Viçosa, Minas Gerais, Southeastern Brazil: Dendrologia (20° 46’21”S 42° 52’25”W), *Recanto das Cigarras* (20° 45’26”S 42°51’45”W) and *Horto Botânico* (20° 45’25”S 42°52’23”W). The first two areas are fragments of secondary Atlantic forest while *Horto Botânico* is a living plant collection comprising native and exotic plant specimens. The fungus garden of each colony was transferred to a plastic pot (500 ml) and then placed in a plastic tray (43 × 29 × 7 cm). In the base of the pots, we made a 2 cm diameter exit hole to allow the passage of workers for the foraging arena in the tray. The inner sides of each tray were covered with neutral talcum powder (magnesium silicate) to prevent the ants from escaping. We offered fresh leaves of *Acalypha wilkesiana* (Euphorbiaceae) daily as forage for the ants. The colonies were maintained under controlled temperature (25 ± 2°C) and humidity conditions (75 ± 3% RH). In order to check if *Escovopsis* and *Escovopsioides* were naturally present in the collected colonies, we sampled fragments from fungus gardens and plated these on growth media. The presence of these fungi was recorded and is presented in the Supplementary Material. *Escovopsis* was found in five of the twelve colonies while *Escovopsioides* was found in just one, and the colonies were considered suitable for conducting the experiment since they were foraging normally.

In both experiments, the same fungal isolates were used: *Escovopsis moelleri* (VIMI-10.0001; CBS 135748), *Escovopsioides nivea* (VIMI-17.0142; MZ097362, MZ097363, and MZ097364)) and *Trichoderma longibrachiatum* (VIMI-17.0135; MZ097365). In the paired culture bioassay (*in vitro*) we also used a *Leucoagaricus gongylophorus* isolate (VIMI-17.0137). *Trichoderma longibrachiatum* was used in the assays for comparison with *E. moelleri* and *E. nivea*. This allowed us to evaluate if the possible effects observed in our experiments is due to *E. moelleri* and *E. nivea* or can be caused by the presence of any other fungi. The genus *Trichoderma* constitutes a group close to *Escovopsis* and *Escovopsioides*, belonging also to the family Hypocreaceae, and can be found in leaf-cutting ant colonies (Rocha et al., 2014, 2017; Montoya et al., 2016).

*Escovopsis moelleri* was obtained from the fungus collection of the Laboratory of Insect-Microorganism-Interactions, Universidade Federal de Viçosa (LIIM-UFV). This isolate was collected from the fungus garden of *Acromyrmex subterraneus molestans* (Augustin et al., 2013) and stored on silica gel at 5°C. *Leucoagaricus gongylophorus*, *E. nivea*, and *T. longibrachiatum* (all isolated from this study by D.M.F.M.) were collected from *A. subterraneus subterraneus* fungus gardens, the last one from the fungus garden of a dead colony kept in the laboratory.

*Leucoagaricus gongylophorus*, *E. nivea* and *Escovopsis moelleri* were cultivated on MEA (20 g malt extract and 15 g agar l^–1^), while *T. longibrachiatum* was cultivated on PDA 20% (7.8 g PDA, KASVI^®^ plus 12 g agar l^–1^). Subsequently, these fungi were incubated at 25°C for 7 days. After this period, they were re-isolated (ca. 2 times) until we obtained a pure culture. The isolate of *L. gongylophorus* was identified through its morphological characteristics, such as the production of gongylidia. *Escovopsioides nivea* and *T. longibrachiatum* were identified by their morphological characteristics and at the molecular level. For morphological identifications, we made microscope slides. Preparation of microscope slides was carried out from plates containing fungi grown on plates with culture medium. Plates were initially observed under a stereoscopic dissecting microscope, so using a sterile needle we could pick up regions with conidiophores and parts of the *Leucoagaricus* colony to observe the presence of gongylidia. A drop of lactoglycerol or lacto-fuchsin was placed on slides and then we placed the fungal material. With the help of two needles, we disentangled the material, if necessary. We placed the cover slip on top of this. The structures were observed with a Nikon (Eclipse E200) light microscope. The molecular characterization was conducted by sequencing the genomic region translation elongation factor (*tef*). The obtained sequences were compared with other sequences available at GenBank through Basic Local Alignment Search https://blast.ncbi.nlm.nih.gov/Blast.cgi). Further details of the molecular identifications are available in the Supplementary Material. The isolates were stored in 10% glycerol at −80°C and included in the Laboratory of Insect-Microorganism-Interactions mycological collection.

### Experiment I (*in vivo*) – Impact of Fungi at Different Levels of Complexity on Colonies of Leaf-Cutting Ants

The aim of this experiment was to evaluate the impact caused by *Escovopsis* and *Escovopsioides* in colonies of leaf-cutting ants divided into different levels of colony complexity. For this, we exposed queenright colonies, queenless colonies and fungus gardens to *Escovopsis* and *Escovopsioides*, individually.

#### Experimental Setup

To prepare the conidial suspensions, we first grew the isolates of *E. moelleri* and *E. nivea* on plates containing MEA, while *T. longibrachiatum* was grown on PDA 20%. These fungi were incubated at 25°C for 15 days. After this period, fragments of fungus were removed from the plates and individually inserted into a Falcon tube containing sterile distilled 0.01% of Tween 80^®^ + NaCl 0.85% solution. The suspensions were stirred for 3 min and then filtered using sterile gauze. This procedure allowed the separation of the conidia from hyphal fragments, resulting in suspensions containing only conidia. We prepared the suspensions according to the maximum conidial concentrations we could obtain for each fungus. The concentrations of the suspensions were determined using a Neubauer chamber. *Escovopsis moelleri* and *T. longibrachiatum* suspensions contained 1 × 10^8^ conidia ml^–1^ while *E. nivea* suspension contained 1 × 10^6^ ml^–1^. These were the greatest concentrations we could obtain for each fungus. All suspensions were kept at 5°C overnight.

**FIGURE 2:**
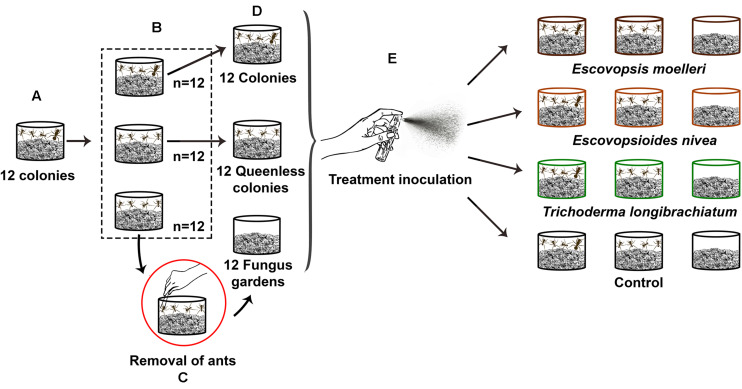
Schematic representation of experimental set-up. Twelve colonies of the leaf-cutting ant *Acromyrmex subterraneus subterraneus* were necessary to assemble the experiment. **(A)** Each of these 12 initial colonies was separated in three fragments. **(B)** One fragment consisted of a queenright colony (fungus garden + workers + queen), and the two other fragments consisted of a queenless colony (fungus garden + workers, without queen), resulting in the total of 12 queenright colonies and 24 queenless colonies. Each queenright colony with their respective queenless colonies were placed in 250 ml individual pots and maintained together in the same tray for 30 days to allow the growth of the fungus gardens. **(C)** After this period, in order to obtain the fungus gardens without any ants, we removed—using tweezers—the workers from 12 queenless colonies. **(D)** Thus, we obtained our three colonies complexity levels: queenright colony (*n* = 12), queenless colony (*n* = 12), and fungus garden (*n* = 12). **(E)** We exposed each of these colonies complexity levels to one of the three fungal treatments or control: conidial suspension of *Escovopsis moelleri* (1 ×10^8^ conidia ml^– 1^), *Escovopsioides nivea* (1 ×10^6^ conidia ml^– 1^), *Trichoderma longibrachiatum* (1 ×10^8^ conidia ml^– 1^), or control (0.01% Tween 80^®^ + NaCl 0.85% solution). The inoculation of suspensions was done through a sterile spray bottle. We sprayed 4 ml of conidial suspensions or the control on the surface of fungus gardens. The queenright colonies, queenless colonies and fungus gardens that originated from the same initial colony were exposed to the same treatment. There were three replicate (sub-)colonies per treatment (combination of a fungus treatment + a colony complexity level).

In order to verify the effects of the three different levels of colony complexity, we performed the following procedure: each initial colony was divided into three fragments (Figure 2A), one fragment consisted of a queenright colony and the two remaining fragments consisted of queenless colonies, totalling 12 queenright colonies and 24 queenless colonies (Figure 2B). Each fragment was placed in 250 ml plastic pots (Figure 2B). The fragments were maintained in the separate pots in the same tray for 30 days; by this time the fungus garden fragment reached the top of the 250 ml pot. To obtain the treatment that consisted only of fungus garden, we carefully removed the ants (eggs, larvae, pupae and adults) from 12 queenless colonies using forceps 1 day before the start of the experiment (Figure 2C). For this, the fungus gardens were fragmented to get access to all their regions so we could remove all the ants. We observed in preliminary tests that fungus gardens decreased in weight and suffered changes in their physical structure when the ants were removed, presumably due to the manipulation during the procedure. In order to apply the same conditions to all fragments, the fungus gardens of the 12 queenright colonies and 12 queenless colonies were fragmented similarly, without damaging the queens or the workers. This step was important also to observe if queenright colonies were indeed queenright and queenless colonies were queenless. We opted not to use insecticides to remove the ants from fungus gardens as some insecticides may have inhibitory effects on fungal growth (Cowley and Lichtenstein, 1970; Olmert and Kenneth, 1974; Ali et al., 2012), which could interfere in our experiment. Thus, we considered mechanical removal the most adequate procedure. Subsequently, each pot that contained each complexity level was placed individually in a tray (343 × 200 × 66 mm). The inner sides of the trays were covered with neutral talcum powder to prevent the ants from escaping. Thus, we had in total 12 queenright colonies (fungus garden + queen + workers), 12 queenlees colonies (fungus garden + workers) and 12 pots containing only fungus garden (Figure 2D). To keep the humidity of queenright colonies, queenless colonies, and fungus gardens, we placed a piece of cotton wool moistened with water under the pots. To assemble and conduct the experiment many steps were required and are listed in Supplementary Table 1.

We inoculated 4 ml of conidial suspensions of *E. moelleri* (1 × 10^8^ conidia ml^–1^), *E. nivea* (1 × 10^6^ conidia ml^–1^) and *T. longibrachiatum* (1 × 10^8^ conidia ml^–1^) individually, on each pot (Figure 2E). For the control blank, we inoculated 4 ml of 0.01% Tween 80^®^ + NaCl 0.85% solution (Figure 2E). The queenright colonies, queenless colonies and fungus gardens that originated from the same initial colony were exposed to the same treatment. Each combination of fungus treatment with a colony complexity level was replicated three times. The inoculation of conidial suspensions was done using a sterile spray bottle (Figure 2E). We sprayed the suspensions on the surface of fungus gardens until all 4 ml were dispensed (Figure 2E). The trays that contained each treatment were placed randomly in three shelves. Each shelf contained one repetition of each treatment. During the experiment, laboratory conditions were 25 ± 2°C, 75 ± 3% RH and 12 h photoperiod.

#### Data Collection

The experiment was conducted blind. The colonies were identified by numbers during the evaluation and we only checked the identities of the treatments at the end of evaluations. Additionally, we allocated the colonies to treatments by drawing lots.

The survival of queenright colonies, queenless colonies and fungus gardens was checked every day. These records were made for 118 days after fungal inoculation. We considered fungus gardens without ants to be dead when they were completely covered by other fungi. The queenright colonies and queenless colonies were considered dead when they did not contain any live ants, their fungus gardens presented a dry texture—almost crumbling—or if they were overgrown by other fungi. We kept the colonies for 7 months after the end of the experiment to see if they remained alive.

To evaluate if there was variation in the weight according to each complexity level or if all of them succumbed to fungal treatment after inoculation, the weights of queenright colonies, queenless colonies and fungus gardens from each treatment were measured. We weighed the queenright colonies and queenless colonies 2 days before the experiment start while the fungus gardens were weighed 1 day before. We repeated this procedure 48 h after inoculations of conidial suspensions and then every 72 h for 20 days. The initial weight of each queenright colony presented some variation and this was also observed between the queenless colonies and among the fungus gardens. These variations are natural since each colony presents particular characteristics and thus it is difficult to find colonies with equal weights.

Middens from each queenright colony and from queenless colonies were weighed to evaluate if ants produced different quantities of midden depending on complexity level and fungal treatment. We weighed the midden produced 24 h after fungal inoculation and then every 72 h for 20 days.

The weights of leaves cut by workers were determined to evaluate if ants altered the amount of food supplied to the fungal cultivar due to the possible detrimental effects of the fungal treatments and the absence of queen (in the case of queenless colonies). For this purpose, we offered, daily, 3 g of fresh *Acalypha wilkesiana* leaves and after 24 h, we weighed the leaves that were not cut (leaves remaining in the trays) for 20 days. To calculate the real quantity of leaves that were cut, we considered the percentage of water loss of these leaves. Thus, we daily maintained on each shelf that had the treatments, one tray containing only fresh *A. wilkesiana* leaves (3 g) that were also weighed in order to evaluate the water loss. Therefore, we calculated the weight of leaves that were cut according to the formula adapted from Antunes and Della Lucia (1999) and Gandra (2014) (below). In the equation Cr refers to the weight of cut leaves, QFi represents the quantity of leaves that we offered, QFf the quantity of leaves that were not cut by ants, and %PA percentage weight of water loss.

Cr=QFi-QFf-%PA

We sampled the fungus gardens from each queenright colony, queenless colony and fungus garden without ants to verify the presence of *Escovopsis*, *Escovopsioides* or *Trichoderma*. We also sampled the middens produced by queenright colonies and queenless colonies to observe if the ants were removing these fungi from their fungus gardens (Supplementary Material). This sampling was always carried out after weighing the midden.

#### Data Analysis

We compared colony survivals related to three complexity levels (queenright and queenless colonies and fungus garden) and to fungal treatment (*E. moelleri*, *E. nivea*, *T. longibrachiatum*, or blank control), using survival analyses with a Weibull hazard distribution (Crawley, 2013). We adjusted a full model with treatment and complexity levels, and an interaction term between these variables. The full model was simplified by deletion of non-significant effects. The colony of origin was included as a frailty factor (Rondeau et al., 2012) in the survival function. The frailty factor is an extension of survival models, which allows adding random factors to this type of model. In this case, the colony of origin was included as a random factor to account for the possibility of pseudoreplication. Analysis was performed in R software (R Core Team, 2020) with survival package v. 2.38 (Therneau and Thomas Lumley, 2015).

We adjusted linear mixed models (LMM) with normal distributions and random intercepts to evaluate the significance of explanatory factors: the colony complexity levels, fungal treatments and time. The distribution was evaluated based on the residuals of the model and we opted for the normal adjustment. The response variable consisted of: (i) weight of queenright colonies, queenless colonies, fungus gardens and weight of all these complexity levels together submitted to fungal treatment; (ii) midden weight produced by queenright colonies and queenless colonies and weight of all of them related to fungal treatment and (iii) weight of leaves cut by ants from queenright colonies and queenless colonies and their weight associated with fungal treatment. Response variables were analyzed in separate models. For each response variable we adjusted a full model with treatment and complexity levels, and interactions between treatment and time, as well as complexity levels and time of evaluation. The identity of each original colony and pot was considered as a random factor in analyses. The full model was simplified by deletion of non-significant effects. The models were compared using Chi-squared test (χ^2^) (*P* < 0.05). We performed all analyses in R Software (R Core Team, 2020).

### Experiment II (*in vitro*) – Interaction of Fungi With the Fungal Cultivar of Leaf-Cutting Ants

The aim of this experiment was to evaluate the interaction between *L. gongylophorus* with *Escovopsis*, *Escovopsioides* and *Trichoderma in vitro*, that we considered the simplest level of complexity. Our idea was to verify whether virulence can be different when analyzed from experiments using only the fungi *in vitro*. We evaluated the growth of *L. gongylophorus* through a paired culture assay (co-culture) in Petri dishes with *Escovopsis*, *Escovopsioides*, *Trichoderma*, and blank control.

#### Experimental Setup

The isolates of *E. moelleri*, *E. nivea*, and *T. longibrachiatum* were previously grown in Petri dishes (9 cm in diameter) containing MEA and incubated at 25°C for 10 days. The isolate of *L. gongylophorus* was previously grown on MEA for 15 days, because it has slower growth. After this period, 8 mm diameter disks of *L. gongylophorus* were cut and plated 5 mm from the border of Petri dishes (90 × 15 mm) containing 20 ml of MEA. The plates were incubated at 25°C for 15 days, the period required for the growth of this fungus, as described above. After this, mycelium disks of *E. moelleri*, *E. nivea*, and *T. longibrachiatum* of 8 mm diameter were placed individually in plates containing the disk of *L. gongylophorus*. These fungi were inoculated on the opposite side of the fungal cultivar, also at 5 mm from the plate edge. For the control treatment, we used an 8 mm diameter disk of plain MEA instead of the fungal cultivar. Thus, we obtained the four following combinations: (i) *L. gongylophorus* × *E. moelleri* (*n* = 10), (ii) *L. gongylophorus* × *E. nivea* (*n* = 10), (iii) *L. gongylophorus* × *T. longibrachiatum* (*n* = 10), (iv) *L. gongylophorus* × control (*n* = 10). During the experiment, the plates were maintained incubated at 25°C and distributed on five shelves of an incubator. Each shelf contained two blocks with one repetition of each treatment per block. The growth of fungi was evaluated every 12 h, for 10 days. However, we conducted the analysis using the data of day 5, when the first fungal isolate reached the fungal cultivar on the opposite side of the plate. We photographed and scanned the plates using a digital camera (Nikon D 2000) and a multifunction printer (HP LaserJet Pro CM1415fnw). From the images, we measured the growth of fungal cultivar *L. gongylophorus* using ImageJ 1.49v software.

#### Data Analysis

To compare the growth of the *Leucoagaricus gongylophorus* isolate in the presence of *Escovopsis moelleri*, *Escovopsioides nivea*, *Trichoderma longibrachiatum* fungi, and control (agar), we adjusted linear mixed models (LMM) with normal distributions and random intercepts. For this, we considered presence of fungi (*E. moelleri*, *E. nivea*, and *T. longibrachiatum* and control) and time as explanatory factors, and colony radius (cm) of *L. gongylophorus* as a response factor. The identity of each plate was considered as a random factor in both analyses. We then tested the interaction between the explanatory factors. Also, we compared *L. gongylophorus* growth, performing multiple comparisons with multcomp package v. 1.4–13 (Hothorn et al., 2016), to verify the potential differences of *L. gongylophorus* growth in the presence of *E. moelleri*, *E. nivea, T. longibrachiatum* and control before inoculation of fungi and control (after 15 days of *L. gongylophorus* inoculation) and at the end of the experiment (after 108 h). We performed all analyses in R Software (R Core Team, 2020).

## Results

### Experiment I (*in vivo*) – Survival of Colonies Divided in Complexity Levels and Exposed to Different Fungal Treatments

**FIGURE 3:**
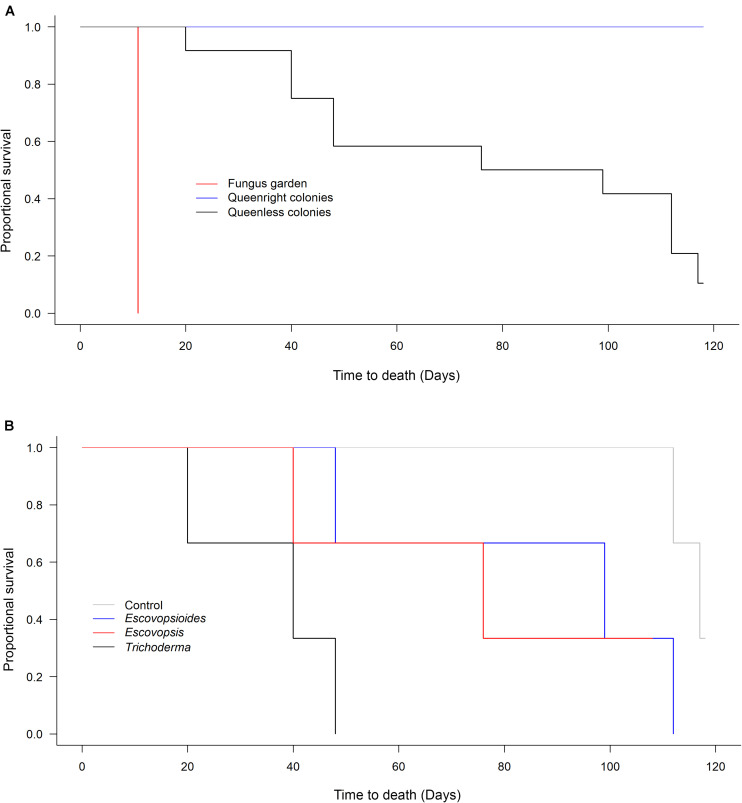
**(A)** Survival of *Acromyrmex subterraneus subterraneus* leaf-cutting ant queenright colonies (blue line), queenless colonies (black line) and fungus gardens (red line) exposed to fungal treatments: conidial suspension of the fungi *Escovopsis moelleri*; *Escovopsioides nivea*; *Trichoderma longibrachiatum*; blank control (0.01% Tween 80^®^ solution + saline solution - NaCl 0.85%). Each treatment was replicated three times (combination of a fungus treatment + a colony complexity level). Survival was checked every day from the day of inoculation with fungal treatments (Day 0) until 118 days post-inoculation. The fungus gardens were considered dead when they were completely covered by other fungi and presented a dry texture. We considered the queenright colonies and queenless colonies dead when they did not contain any live ants. **(B)** Survival of *Acromyrmex subterraneus subterraneus* leaf-cutting queenless colonies exposed to *Escovopsis moelleri*; *Escovopsioides nivea*; *Trichoderma longibrachiatum* or blank control (0.01% Tween 80^®^ solution + saline solution – NaCl 0.85%). From the analyses comparing survival between the three complexity levels, we observed that only queenless colonies suffered variation in mortality over time **(A)**. For this reason, we compared within this level the effect of each fungal treatment individually **(B)**. There was no difference between queenless colonies inoculated with *E. nivea*, *T. longibrachiatum* and blank control (χ^2^_[1]_ = 1.868, *P* = 0.393). However, queenless colonies inoculated with *E. moelleri* treatment died faster (χ^2^_[1]_ = 9.582, *P* = 0.002). A survival analysis with a Weibull distribution was conducted and the models compared using χ^2^ test (*P* < 0.05).

All queenright colonies remained alive during the 118 days evaluated. Seven months after the end of the experiment, the queenright colonies were still alive and at this juncture were discarded. At the other extreme, all fungus gardens (without queen and workers) died 11 days after the inoculation of fungal treatments, including the blank control, independent of the fungus that was inoculated. Therefore, between-treatment variation in survival was only observed in the queenless colonies (Figure 3A).

The survival times of queenless colonies did not differ between the blank control (115.66 ± 1.85 days; mean ± S.E.), *E. nivea* (86.33 ± 19.5 days; mean ± S.E.), and *T. longibrachiatum* (74.66 ± 19.6 days; mean ± S.E.) (χ^2^_[1]_ = 1.868, *P* = 0.393; Figure 3B). However, queenless colonies submitted to *E. moelleri* died more quickly (36 ± 8.3 days; mean ± S.E.) than queenless colonies infected with *E. nivea* and *T. longibrachiatum* (χ^2^_[1]_ = 9.582, *P* = 0.002; Figure 3B).

#### Colony Weights

There was an interaction between complexity level and time (χ^2^_[11]_ = 290.8; *P* < 0.001) as well as between fungal treatment and time (χ^2^_[21]_ = 164.5; *P* < 0.001) when considering colony weights. Both complexity level and time were influenced and modified over time according to interaction analyses. These results probably are due to the first 11 days of evaluation (8 evaluations in total) in which different treatments had similar values (mainly in relation to fungal treatments), with greater divergence subsequently occurring between treatments.

**FIGURE 4:**
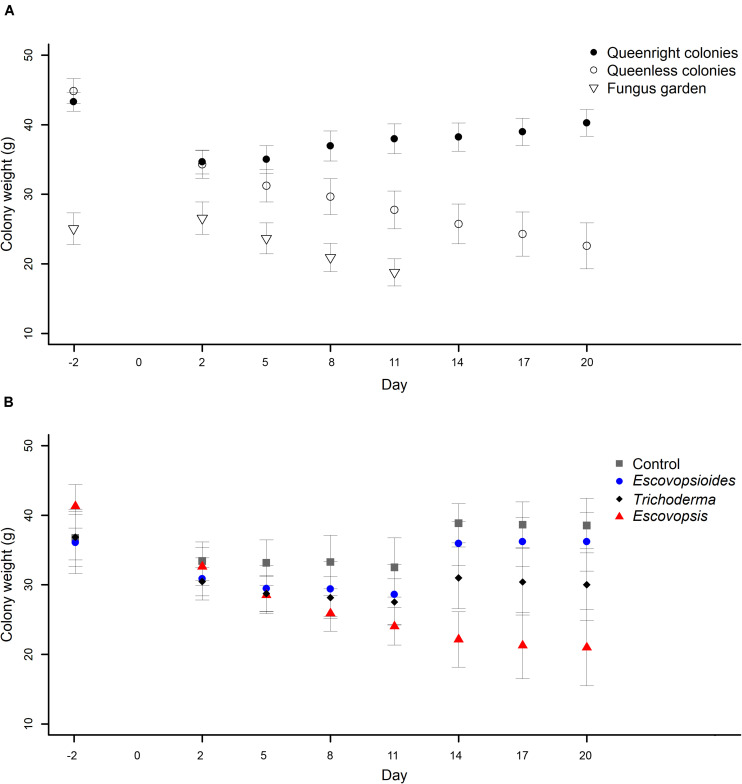
**(A)** Weights of *Acromyrmex subterraneus subterraneus* leaf-cutting ant colonies in the three complexity levels: queenright colonies (closed circle), queenless colonies and (open circle) and fungus gardens (open triangle) exposed to fungal treatments *Escovopsis moelleri*; *Escovopsioides nivea*; *Trichoderma longibrachiatum*) and to blank control (0.01% Tween 80^®^ solution + saline solution - NaCl 0.85%). The weight of queenright colonies and queenless colonies was measured 2 days before inoculation (day -2) while fungus gardens were weighed 1 day before inoculation (day -1). Day 0 represents the day on which we carry out the inoculation of fungal treatments at each colony complexity level. Weighing was repeated 48 h after inoculation of conidial suspensions for all treatments (day 2), and then every 72 h until day 20. The fungus gardens were evaluated until day 11, when all of them were considered dead. There was an interaction between the complexity level of colonies and the days (χ^2^_[11]_ = 290.8; *P* < 0.001), indicating that the difference observed between different complexity levels changes over time. **(B)** Weights of *Acromyrmex subterraneus subterraneus* leaf-cutting ant colonies of three complexity levels exposed to *Escovopsis moelleri* (red triangle); *Escovopsioides nivea* (blue circle); *Trichoderma longibrachiatum* (black diamond) or blank control (0.01% Tween 80^®^ solution + saline solution - NaCl 0.85%) (gray square). Conidial suspensions and blank control were inoculated on day 0 and the queenright colonies and queenless colonies were evaluated until day 20 after inoculation. Weighing was repeated 48 h after inoculation of conidial suspensions for all treatments, and then every 72 h. There was an interaction between the complexity level of colonies and time (χ^2^_[21]_ = 164.5; *P* < 0.001), indicating that the difference observed between fungal treatments undergoes changes over time. Significance was evaluated using χ^2^ test (*P* < 0.05).

We observed that all queenright colonies lost weight the day after the inoculation of conidial suspensions, independent of the fungal treatment to which they were exposed, and then recovered weight over subsequent days (Figure 4A). Queenless colonies showed a similar behavior after fungal inoculation, but the weight continued to drop over time until the end of the experiment (Figure 4A). In contrast, we observed on the first day of evaluation that fungus gardens suffered a small increase in their weight. This is likely to have been due to some change in how hydrated the mycelia were. Over time, the entire fungus garden was consumed and the weight starts to decrease. All fungus gardens died on the eighth day of evaluation (Figure 4A).

In relation to fungal treatments, all the complexity levels submitted to *Escovopsis* suffered a weight reduction over time (Figure 4B). This result was not observed in any other treatment. Queenright colonies, queenless colonies and fungus gardens inoculated with the blank control, *E. nivea* and *T. longibrachiatum* suffered a similar initial reduction in weight to those inoculated with *Escovopsis*, but by evaluation day 8 after fungal inoculation, they began to recover.

#### Midden Weights

**FIGURE 5:**
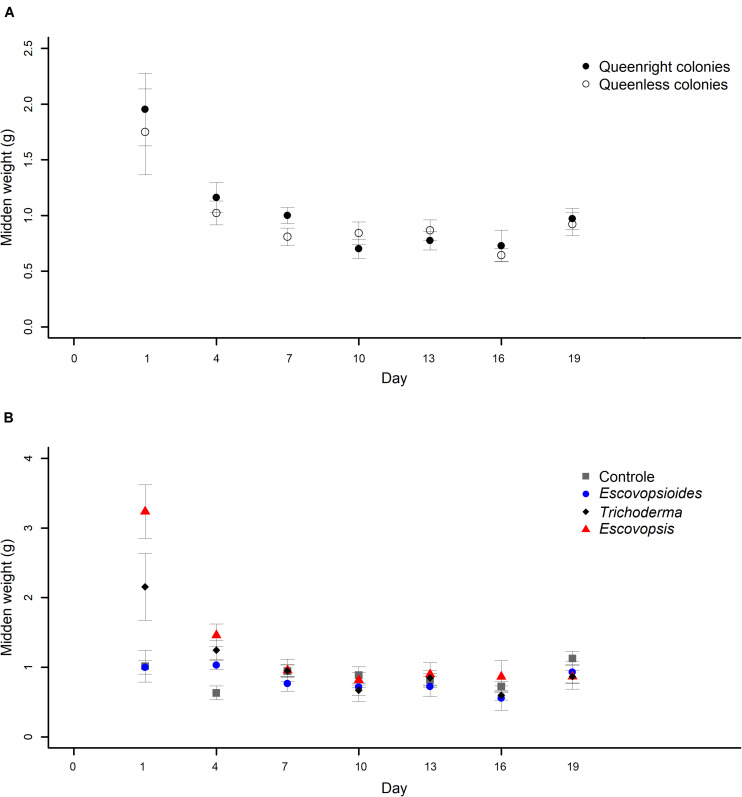
**(A)** Weights of midden produced by *Acromyrmex subterraneus subterraneus* leaf-cutting ant queenright colonies (closed circle) and queenless colonies (open circle) exposed to three fungal treatments and to blank control. To conduct the analysis, we adjusted linear mixed models (LMM) considering each sample as a repeated measure. The weight of midden was measured 24 h after inoculation of conidial suspensions for all treatments (day 1) and then, this procedure was repeated every 72 h. The differences between treatments were evaluated by comparing the complete and simplified models. There was no difference in midden production between queenright colonies and queenless colonies (χ^2^_[1]_ = 0.773, *P* = 0.379). **(B)** Weights of midden produced by *Acromyrmex subterraneus subterraneus* leaf-cutting exposed to one of three treatments or to blank control: conidial suspension of the fungi *Escovopsis moelleri* (red triangle); *Escovopsioides nivea* (blue circle); *Trichoderma longibrachiatum* (black diamond); blank control (0.01% Tween 80^®^ solution + saline solution - NaCl 0.85%) (gray square). Conidial suspensions and blank control were inoculated on day 0 and the weight of midden produced by the queenright colonies and queenless colonies was evaluated until day 19 after inoculation. The weight of midden was measured 24 h after inoculation of conidial suspensions for all treatments (day 1) and then, this procedure was repeated every 72 h. To conduct the analysis, we adjusted linear mixed models (LMM) considering each sample as a repeated measure. There was an interaction between fungal treatments and days (χ^2^_[18]_ = 102.84; *P* < 0.001), indicating that the fungal treatments affected midden production in different manners over time. Significance was evaluated using χ^2^ tests (*P* < 0.05).

There was no interaction between complexity level and time (χ^2^_[6]_ = 5.251; *P* = 0.512; Figure 5A). However, there was an interaction between fungal treatment and time (χ^2^_[18]_ = 102.84; *P* < 0.001; Figure 5B).

The weights of middens produced by ants in queenright and queenless colonies inoculated with fungal treatments and blank control did not differ from each other over time (χ^2^_[1]_ = 0.773, *P* = 0.379; Figure 5A). On the first day of evaluation (24 h after inoculation), both produced a large amount of midden which decreased and remained stable until the end of the experiment.

Midden production was high on the first day after inoculation of *E. moelleri* and was higher in this treatment than in the blank control, *E. nivea*, and *T. longibrachiatum* (Figure 5B). Over time, midden production was similar, independent of the fungus applied, except on the second day of evaluation, on which queenright and queenless colonies of the control group presented less midden production than the fungal treatments (Figure 5B).

#### Weights of Leaves Cut by Ants

There was an interaction between the two complexity levels (queenright and queenless) and the days of evaluation (χ^2^_[19]_ = 30.161; *P* = 0.049). We also found an interaction between fungal treatment and time (χ^2^_[57]_ = 77.863; *P* = 0.034).

**FIGURE 6:**
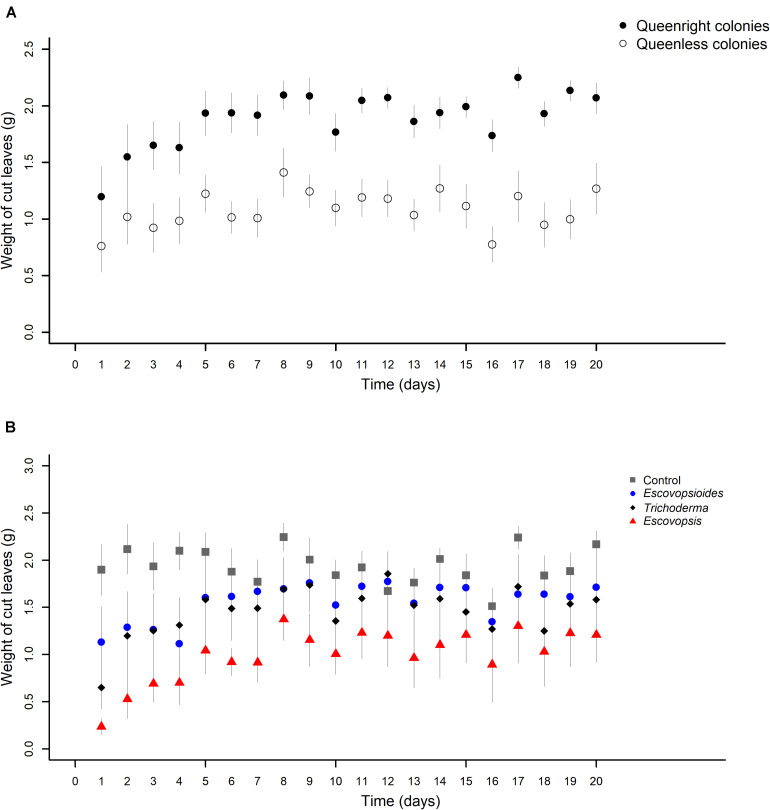
**(A)** Weights of leaves cut by *Acromyrmex subterraneus subterraneus* leaf-cutting ant queenright colonies (closed circle) and queenless colonies (open circle) exposed to three fungal treatments and blank control. The weight of cut leaves was measured every day after inoculation of conidial suspensions for all treatments (day 1) until day 20 after inoculation. To conduct the analysis, we adjusted linear mixed models (LMM) considering each sample as a repeated measure. There was interaction between complexity levels and days (χ^2^_[19]_ = 30.161; *P* = 0.049), indicating that the different complexity levels affect the cut of leaves of distinct way over time. **(B)** Weights of leaves cut by *Acromyrmex subterraneus subterraneus* leaf-cutting ant exposed to one of three treatments or to blank control: conidial suspension of the fungi *Escovopsis moelleri* (red triangle); *Escovopsioides nivea* (blue circle); *Trichoderma longibrachiatum* (black square); blank control (0.01% Tween 80^®^ solution + saline solution - NaCl 0.85%) (gray square). Conidial suspensions and blank control were inoculated on day 0 and the weight of cut leaves by ants of the queenright colonies and queenless colonies was evaluated until day 20 after inoculation. The weight of cut leaves was measured daily after inoculation of conidial suspensions for all treatments. To conduct the analysis, we adjusted linear mixed models (LMM) considering each sample as a repeated measure. There was an interaction between complexity levels and time (χ^2^_[57]_ = 77.863; *P* = 0.034), indicating that the fungal treatments cause different effects in the cut of leaves over time. The significance was evaluated using χ^2^ tests (*P* < 0.05).

In the first 2 days after exposure to fungal treatments, there was a similarity in the weights of leaves cut by queenright and queenless colonies. From the third day, queenright colonies started to cut a larger amount of leaves than queenless colonies (Figure 6A). Probably because of this variation over time, we observed an interaction between the complexity levels and time.

In relation to fungal treatments, in general, all the complexity levels inoculated with *Escovopsis* cut less leaves than those inoculated with other fungi or blank control over the 20 days (Figure 6B). There was a fluctuation within each treatment, but this did not change the main pattern.

### Experiment II (*in vitro*) – Interaction of Fungi With the Fungal Cultivar of Leaf-Cutting Ants Paired Culture Bioassay

**FIGURE 7:**
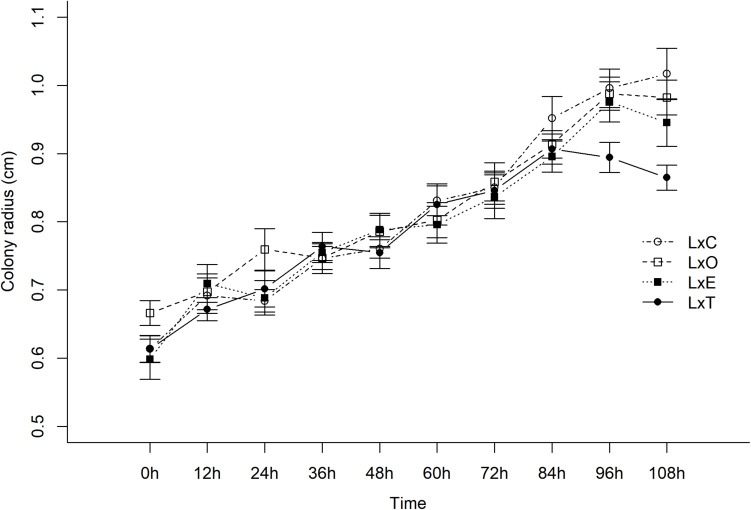
Growth of *Leucoagaricus gongylophorus* (L) in paired culture bioassays with *Escovopsis moelleri* (E), *Escovopsioides nivea* (O), *Trichoderma longibrachiatum* (T) fungi, or control (C). We adjusted linear mixed models (LMM) with normal distributions and random intercepts. There was a significant effect of fungal treatment and time. The *in vitro* growth of *L. gongylophorus* was different in the presence of *E. moelleri*, *E. nivea*, *T. longibrachiatum*, and control over time (χ^2^_[3]_ = 13.81, *P* = 0.003). There was no difference between the growth of *L. gongylophorus* in the presence of control and *E. nivea* (0.98 ± 0.02 cm) (z = 1.0, *P* = 0.31) after 108 h. Nevertheless, in the presence of *T. longibrachiatum* (0.86 ± 0.01 cm) (mean ± S.E.) and *E. moelleri* (0.94 ± 0.03 cm), the colony radius of *L. gongylophorus* was smaller than the colony radius observed in controls (1.01 ± 0.03 cm) (*T. longibrachiatum*: z = 4.43, *P* < 0.001; *E. moelleri*: z = 2.03, *P* = 0.04).

The growth of *L. gongylophorus in vitro* varied with the presence of *E. moelleri*, *E. nivea, T. longibrachiatum* and control over time (χ^2^_[3]_ = 13.81, *P* = 0.003, Figure 7). Before the inoculation of other fungi on the plates (time 0), there was no difference in growth of *L. gongylophorus* among plates of the control and *E. moelleri* (z = −0.43, *P* = 0.66), *E. nivea* (z = 1.53, *P* = 0.12) and *T. longibrachiatum* (z = −0.006, *P* = 0.99; Figure 7). After 108 h, the colony radius of *L. gongylophorus* in the presence of *T. longibrachiatum* (0.86 ± 0.01 cm; mean ± S.E.) and *E. moelleri* (0.94 ± 0.03 cm) was smaller than the colony radius observed in control (1.01 ± 0.03 cm) (*T. longibrachiatum*: z = 4.43, *P* < 0.001; *E. moelleri*: z = 2.03, *P* = 0.04). However, there was no difference between the growth of *L. gongylophorus* in the presence of control and *E. nivea* (0.98 ± 0.02 cm) (z = 1.0, *P* = 0.31).

## Discussion

Our objective was to investigate whether the parasitic fungus *Escovopsis moelleri* and the antagonist *Escovopsioides nivea* represent threats to the health of leaf-cutting ants’ colonies. We also tested whether the virulence level of these fungi varied according to levels of colony complexity. The *in vivo* experiment showed that although the queenright colonies exposed to *E. moelleri* suffered a reduction in their weight, this was not enough to compromise their survival. This indicates that *Escovopsis* may not always present a high virulence to colonies as has been previously suggested (Currie et al., 1999a; Currie, 2001). Augustin (2011) also found that *Escovopsis microspora* and *E. nivea*, in high conidia concentrations, did not cause the death of *Atta sexdens rubropilosa* colonies, adding weight to the generality of our conclusion. We hypothesized that workers may control the growth of these fungi inside the colonies through their defence mechanisms. Some of these mechanisms are already well-known, such as the production of antimicrobial compounds by the metapleural glands (Bot et al., 2002; Poulsen et al., 2002; Fernández-Marín et al., 2006, 2009; Yek et al., 2012), hygienic behaviors such as grooming and weeding (Currie and Stuart, 2001) and a symbiotic association with a filamentous bacterium (actinobacteria) that secretes antimicrobial substances (Currie et al., 1999b). In ants of the genus *Acromyrmex*, an association with the actinomycete bacterium *Pseudonocardia* has been demonstrated, in which cultures of *Pseudonocardia* are maintained on the ant cuticle and produce antifungal metabolites. These are used by the ants to help defend the fungus garden from *Escovopsis* (Poulsen et al., 2010) and other fungi (Dângelo et al., 2016; Goldstein and Klassen, 2020). In relation to the queenless colonies, most suffered a decrease in their weight leading to their subsequent death, independent of the fungal treatment or control. Some studies have shown that the queen is not only important for reproduction but can also influence colony cohesion (Vienne et al., 1998; Della Lucia et al., 2003). According to Sousa-Souto and Souza (2006), the absence of the queen in a colony of leaf-cutting ants *Atta sexdens rubropilosa* increased the workers’ mortality and decreased the refuse disposal, that could indicate a disruption in colony’s internal tasks. In addition, the workers in the absence of the queen are able to carry out the basic activities of the colony, but with a lower level of organization (Hölldobler and Wilson, 1990). While debate on the role of the queen (vs. workers) as the putative controlling force in social insect colonies has focused mostly on chemical communication and reproduction (e.g., Keller and Nonacs, 1993; Olejarz et al., 2017; Villalta et al., 2018), it could well be that further investigation of other functions such as hygiene bears fruit in future studies. In the present case, the important distinction would be between (a) the absence of the queen as an organizing presence on the workers and (b) the reduced necessity for workers to engage in tasks related to reproduction so different time budgets (although one might expect this to *increase* hygiene).

Considering this and based on our results, it seems that the absence of the queen naturally causes a negative impact on queenless colonies. As demonstrated here, *E. moelleri* promoted a great decrease in the weight of colonies. In this manner, since the queenless colonies seem to be already debilitated, probably rendering them limited resources, it is possible that a better strategy of *E. moelleri* would be to increase the exploitation of its host resource, reflected in an increase in virulence. This strategy may allow an early parasite reproduction and transmission to new hosts in a natural setting.

Similar to queenless colonies, the fungus gardens without ants also diminished in weight. However, they died more rapidly, at only 11 days after the inoculation of fungi treatments. It is important to note that we did not observe any fungal sporulation in queenright colonies and queenless colonies. In contrast, *Escovopsis*, *Escovopsioides*, *Trichoderma*, and other fungi were observed growing in fungus gardens without ants. These results indicate that the ants are very important to the protection of a colony against parasites as the fungal cultivar seems not to defend itself alone. Thus, the absence of ants probably rendered the fungus gardens more susceptible to infection. Our results clearly demonstrated that ant-free fungus gardens represent a completely unrealistic situation. In addition, these results are in line with other studies that showed the growth of alien fungi and signals of infection in fungus gardens not tended by workers (Currie et al., 1999a; Augustin, 2011; Wallace et al., 2014; Kellner et al., 2017).

The protection of the fungal cultivar by leaf-cutting ants can be performed through hygienic behaviors as fungus grooming and weeding (Currie and Stuart, 2001). Fungus grooming is characterized by the removal of parasite spores from the fungus gardens while weeding is the removal of infected garden pieces or of vegetal material contaminated (Currie and Stuart, 2001). The infected material is discarded in the midden, located in specific underground chambers or outside the colony (Hölldobler and Wilson, 2009; Lacerda et al., 2011). The removal of contaminants from the fungus gardens is very important to control infections and consequently the maintenance of colony health. In our study, we found that queenright and queenless colonies responded similarly to the presence of alien fungi on their fungus gardens in terms of midden production. In both of these colonies’ complexity levels, the amount of midden produced by workers was high on the first day post-inoculation of *E. moelleri* and *T. longibrachiatum*. We hypothesize that workers tried to remove both fungi from their fungus gardens to control its growth soon after the inoculation of the fungi. Possibly, this initial effort does not completely remove the inoculated fungi, but it can guarantee a control of these fungi so that the colony resumes other activities without any major damage. On the other hand, both queenright and queenless colonies exposed to control and *E. nivea* maintained the production of midden unaltered throughout the evaluation of the experiment. This may indicate that workers do not put much effort into removing *E. nivea* from their fungus gardens, probably because this fungus represents an even smaller threat than *E. moelleri* or *T. longibrachiatum*. Alternatively, they may be unable to detect it.

On the day after the inoculation of fungal treatments in the queenright colonies, workers cut smaller amounts of leaves than in the following days. We suspect that the workers initially invested time and effort removing the parasites from their queenright colonies rather than cutting leaves to be incorporated into the fungus gardens. This result seems to be supported by the results of midden production, because on this same day the amount of midden produced by colonies (mainly those inoculated with *E. moelleri* and *T. longibrachiatum*) was high, as discussed above. After this period, the queenright colonies exposed to fungi increased the amount of cut leaves. It is possible that workers increase the cutting of vegetal material to incorporate them in the fungus gardens, promoting the fungal cultivar grow. Similar results were found by Augustin (2011) when colonies of *Atta sexdens rubropilosa* were exposed to *E. microspora* and it was observed that workers increased the incorporation of leaf fragments in the fungus gardens 50 h after fungal inoculation. According to this author, this could act as a mechanism that circumvents the possible negative effects caused by this fungus on the colonies and we believe this same hypothesis can be applied to our results. In contrast, the amount of cut leaves, in queenright and queenless colonies treated with *E. nivea*, were similar to *T. longibrachiatum* and control with some variation over time. This can indicate once again that fungus does not pose a risk to the health of colonies and the ants do not alter the foraging activity to overcome its presence in their fungus gardens.

The results of the *in vitro* bioassay have shown that growth of *L. gongylophorus* is slowed in the presence of *E. moelleri* and *T. longibrachiatum* in paired cultures. We observed that *E. moelleri* may easily overgrow the fungal cultivar, probably because there are no workers to control it in the culture medium. This overgrowth on the fungal cultivar by *Escovopsis in vitro* has also been shown in previous studies (Folgarait et al., 2011a; Varanda-Haifig et al., 2017). The absence of other microorganisms in this interaction, which could compete for the same nutritional resources, may have facilitated its growth as well. In fact, this was observed in our assay after approximately 7 days post-inoculation of *E. moelleri* in the culture medium with the fungal cultivar, in contrast to what we observed in queenright colonies.

The antagonist fungus *E. nivea* did not have any effect on *L. gongylophorus*. Also, we observed that *E. nivea* only overgrew the fungal cultivar after approximately 10 days post-inoculation in the culture medium with *L. gongylophorus*. This result, associated with the data obtained from the *in vivo* experiment, may suggest that this fungus has a low virulence strategy toward the fungal cultivar of leaf-cutting ants. According to Varanda-Haifig et al. (2017) and Osti and Rodrigues (2018), *E. nivea* isolates were capable of inhibiting the growth of *L. gongylophorus* in the culture medium, however, their isolates seem to be less aggressive compared to *Escovopsis* sp. (unidentified isolates) similar to what we observed here.

Our study aimed to investigate the virulence of fungi commonly found in colonies of leaf-cutting ants. From this study, we showed how the virulence of these microorganisms can be influenced by the complexity of interactions that composed a colony. Our results showed that, in general, the fungi *E. moelleri*, *E. nivea*, and *T. longibrachiatum* were not capable of causing the death of queenright colonies of leaf-cutting ants. However, most of the queenless colonies and fungus gardens died, which suggests that the queen and workers are very important to the maintenance of colony health and stability. These results highlight the importance of considering the whole superorganism in studies that investigate the virulence of parasites in colonies of eusocial insects. It is important to point out that *Escovopsis* has been suggested as a potential biological control agent of leaf-cutting ants (Folgarait et al., 2011a, b; Wallace et al., 2014). In this manner, we emphasize the importance of conducting experiments to test this possibility using queenright colonies, since *in vitro* experiments may not represent a realistic approach. In fact, a colony of leaf-cutting ants is not only composed of the fungal cultivar or the queen and workers but is a complex of interactions involving all these organisms and this must be considered.

In addition, we observed that *E. nivea* and *T. longibrachiatum* seem to be less aggressive than *E. moelleri*, and this was observed both *in vivo* and *in vitro*. Our study is one of the first to investigate the impact of *Escovopsioides* on colonies of leaf-cutting ants. This may open new avenues for future research that seek to understand the role of this fungus in the leaf-cutting ants-fungus symbiosis.

To conclude, we propose that *Escovopsis* is far from being the highly virulent parasite it has been cast as in the literature and that this is also true of its sister genus *Escovopsioides*. This would make evolutionary sense if one considers the comparatively long-lived nature of its (superorganism) hosts. Remembering Frank’s (1996) simple definition of virulence (1996) as the harm done by a parasite to its host, there has to date been no study indicating high virulence of either fungi in a healthy and whole colony. Where *Escovopsis* may be virulent, we propose that this is a change in strategy when its host is weakened, which would seem not be true for *Escovopsioides*. A plastic change in virulence strategy such as this could be seen as opportunism.

It is worth including some provisos to our interpretation of our results: (1) although this study is closer to a real field situation than previous studies, it is still a laboratory study with comparatively young colonies while assays of prevalence in the field (cited above) have also focused on younger colonies–we would expect younger colonies to be more vulnerable to parasites; (2) we applied the antagonistic fungi as conidia after holding them overnight at 5°C–this could have affected the abilities of the fungi to establish and infect the colonies, although they were applied at extremely high doses; (3) we used two antagonist species here [although a third was used in Augustin (2011)] so the generality of our conclusions requires testing with more representatives of *Escovopsis* and *Escovopsioides*. It would be interesting to develop new studies that expand on our approach and investigate other factors related to the virulence of these and other fungal species. This could help us to understand the diversity of strategies and virulence these fungi can present, especially as some studies have shown that different strains can vary in their virulence level (Silva et al., 2006; Wallace et al., 2014; Marfetán et al., 2015).

## Data Availability Statement

Raw data and code are available in the Supplementary Material.

## Author Contributions

DM, MC, CM, and SE conceived and designed the research. DM, MC, CM, and GM performed the experiments. TK analyzed the data. DM wrote the original draft. DM, MC, CM, TK, and SE reviewed and edited the manuscript. All authors contributed to the article and approved the submitted version.

## Conflict of Interest

The authors declare that the research was conducted in the absence of any commercial or financial relationships that could be construed as a potential conflict of interest.

## Publisher’s Note

All claims expressed in this article are solely those of the authors and do not necessarily represent those of their affiliated organizations, or those of the publisher, the editors and the reviewers. Any product that may be evaluated in this article, or claim that may be made by its manufacturer, is not guaranteed or endorsed by the publisher.
