# Supplementary material for: Low Virulence of the Fungi Escovopsis and Escovopsioides to a Leaf-Cutting Ant-Fungus Symbiosis
Source: Front Microbiol. 2021 Jul 29;12:673445. doi: 10.3389/fmicb.2021.673445 (PMC8358438; doi:10.3389/fmicb.2021.673445)
Supplement: Supplementary file 1 [file Table_1.pdf]

Supplementary Material

1 Sampling of fungus gardens before the experiments

We conducted sampling of the fungus gardens from the 12 colonies collected to check whether *Escovopsis* and *Escovopsioides* were naturally present. For each colony, we collected ten fragments of fungus garden from three regions: top, middle and base, totalling 30 fragments. For this, we first sampled the top and the base regions of the fungus gardens. Subsequently, each fungus garden was carefully split in half with the aid of a spatula to allow access to the middle region and then we conducted our sampling of this region. The fragments of each region were plated onto Petri dishes containing PDA 20% following incubation at 25ºC for 15 days. The plates were checked daily for the presence of *Escovopsis* and *Escovopsioides* on fragments.

None of the colonies presented *Escovopsis* or *Escovopsioides* growing on the top region of their fungus gardens. We found *Escovopsis* growing in the middle region from only one colony, while *Escovopsioides* was not observed in this region in any of the colonies. The presence of *Escovopsis* and *Escovopsioides* in the base region was detected in four colonies and one colony, respectively. In total, *Escovopsis* was found in five of the 12 colonies and *Escovopsioides* was found in one.

2 Sampling for middens

We also sampled the middens produced by queenright colonies and queenless colonies to observe if the ants were removing these fungi from their fungus gardens. We plated five fragments of midden from each queenright colony and queenless colony on Petri dishes containing PDA 20% and then incubated these at 25°C for 15 days. This procedure was conducted 24 hours after inoculation of conidial suspensions for all treatments (Day 1) and then every 72 hours, totalling 10 days of sampling. We checked the plates daily to verify the occurrence of *Escovopsis*, *Escovopsioides* and *Trichoderma*.

In the same manner as the previous variable (sampling of fungus gardens), we recognized that the ideal would be to identify these fungal genera to species level; this was not possible, however, for practical reasons. No growth of *Escovopsis* and *Escovopsioides* was observed in the midden of queenright or queenless colonies. We suspect that midden could present these fungi, nevertheless, other microorganisms that are also present may have inhibited their growth in the culture medium. On the other hand, *Trichoderma* was found growing the midden from the queenright colonies and queenless colonies, independent of the treatment to which they were exposed.

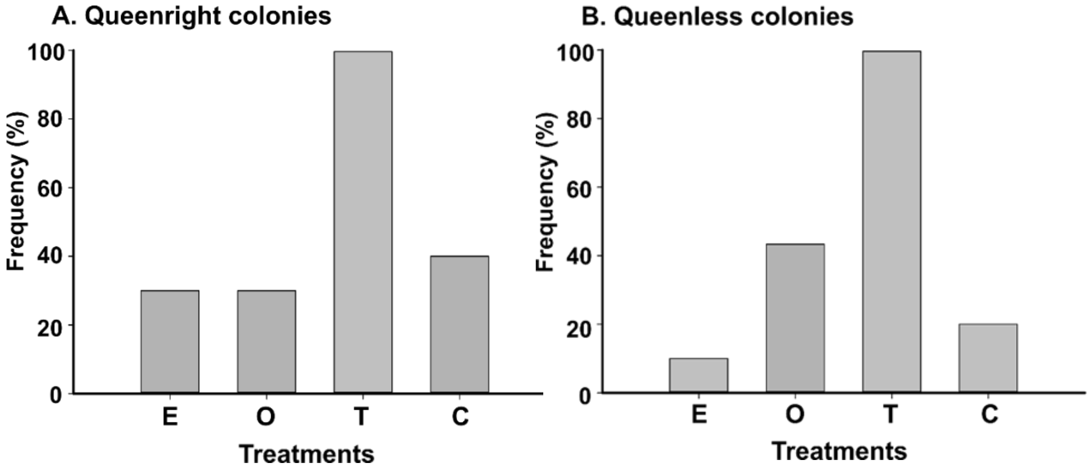

Supplementary Figure 1. Frequency of the fungal genera *Escovopsis*, *Escovopsioides* and *Trichoderma* found in the midden of **(A)** queenright colonies and **(B)** queenless colonies of *Acromyrmex subterraneus subterraneus* leafcutter ants exposed to one of three treatments or to the blank control: conidial suspension of the fungi *Escovopsis moelleri* (E); *Escovopsioides nivea* (O); *Trichoderma longibrachiatum* (T); blank control (0.01% Tween 80® solution + saline solution - NaCl 0.85%; C). The bars represent the mean frequency of presence of these fungi in the 10 days of sampling.

Table 1. Summary of the days and the respective activities conducted for the assembly and evaluation of the experiment

Summary of experiment set up days	
Day –28	Sampling of the colonies' fungus gardens; Division of each initial colony into three fragments (totalling 12 queenright colonies and 24 queenless colonies);
Day –2	Weighing of the 12 queenright colonies and 12 queenless colonies;
Day –1	Removal of ants from the other 12 queenless colonies; Weighing fungus gardens; Preparation of fungal suspensions;
Day 0	Inoculation of fungal treatments; Survival evaluation;
Day 1	Weighing and sampling of midden; Weighing of the leaves cut by ants from queenright colonies and queenless colonies;
Day 2	Sampling of queenright colonies, queenless colonies and fungus gardens;
Day 11	Last day of weighing fungus gardens;
Day 28	Last day of weighing and sampling of midden;
Day 29	Last day of weighing and sampling of queenright colonies and queenless colonies; Last day of weighing the leaves cut by the ants from queenright colonies and queenless colonies;
Day 118	End of the experiment: last day of survival evaluation.

3. Molecular identification

In order to verify the identity of the *Escovopsioides* isolate, this fungus was grown for 10 days in plates containing 100 ml of liquid medium (10 g of sucrose, 2 g L-asparagine, 2 g yeast extract, 1 g KH2PO4, 0.1 g MgSO4.7H2O, 0.44 mg ZnSO4.7H2O, 0.48 mg FeCl3.6H2O and 0.36 mg MnCl2.H2O) (Augustin et al., 2013). DNA was extracted using the Wizard Genomic purification kit protocol, following the manufacturer's instructions. Three genomic regions were amplified by PCR: ITS rDNA (Internal Transcribed Spacer) (White et al. 1990, Schoch et al. 2012), LSU rDNA (Large Subunit) (White et al. 1990, Haugland and Heckman 1998, Currie et al. 2003) and tef1 (Translation

Elongation Factor 1-alpha) (Taerum et al. 2007). Primer pairs and the PCR conditions were as follow: ITS: ITS1-F (5'CTTGGTCATTTAGAGGAAGTAA3') and ITS4-R (5'TCCTCCGCTTATTGATATGC3'). Condition: 5 minutes denaturation at 95°C, 30 cycles consisting of 30 seconds at 95°C, 30 seconds at 56,5°C, 90 seconds at 72°C and final extension step at 72°C for 10 min.

LSU: CLA-F (5' GCATATCAATAAGCGGAGGA 3') and CLA-R (5' GACTCCTTGGTCCGTGTTTCA 3'). Condition: 2 minutes at 95°C, 40 cycles of 30 seconds at 95°C, 60 seconds at 62°C, 90 seconds at 72°C and 5 minutes of extension at 72°C.

tef1: EF1-983F (5'GCYCCYGGHCAYCGTGAYTTYA 3') and EF1-2218R (5' GACTTGACTTCRGTVGTGAC 3'). Condition: 2 min at 95°C, 40 cycles of 30s at 95°C, 60s at 63°C, 90s at 72° and 5 minutes of extension at 72°C.

Reaction results of the ITS region were 23 sequenced in ABI3500 (Life Technologies) and LSU and tef1 regions were sent to Macrogen Inc (dna.macrogen.com/eng/, Seoul, S. Korea) for purification and sequencing. Sequences were attached in contigs with BioEdit v. 7.1.3 (Hall, 1999).

For *Trichoderma* molecular identification, we prepared monosporic cultures from the isolate VIMI 17.0135. The isolate was grown in PDA 20% medium for 7 days in the dark. The mycelium was collected from the agar surface and macerated in liquid nitrogen. Genomic DNA from the sample was extracted using the Wizard® Genomic DNA Purification Kit (Promega Corporation, WI, USA), following the manufacturer's protocol. DNA quality was then analysed with agarose gel electrophoresis (1%) and quantified with Nanodrop 1000ND (Thermo Scientific, USA). We amplified the ITS region, using the primers ITS 1 (5'- TCCGTAGGTGAACCTGCGG - 3') and ITS 4 (5'-TCCTCCGCTTATTGATATGC - 3') (White et al., 1990).

We performed amplifications for the ITS marker in a final volume of 25 µl (12.5 µl of Dream Taq PCR Master Mix Thermo Scientific®; 8.5 µl of water free nuclease; 1 µl of each primer [10 µmol]; 2 µl diluted genomic DNA [25 µl]. The PCR condition was 94 ºC/2 min followed by 35 cycles at 94 ºC/1 min, 55 ºC/1 min and 72 ºC/1 min; and final extension at 72 ºC/5 min. PCR products were purified and sequenced by Macrogen®, South Korea (http://www.macrogen.com). We edited and assembled the sequences with CodonCode Aligner (Codon Code Corporation, 2020). Consensus sequence was compared to homologous sequences using NCBI nucleotide database BLASTn to assess its ID (Altschul et al. 1990).

References:

Altschul, S. F., Gish,W., Miller,W., Myers, E.W., and Lipman, D. J. (1990). Basic local alignment search tool. *J. Mol. Biol.* 215, 403–410. https://doi.org/10.1016/S0022-2836(05)80360-2

Codon Code Corporation (2020). *CodonCode Aligner*. Available online at: http://www.codoncode.com/aligner

Currie, C. R., Bot, A. N. M., and Boomsma, J. J. (2003). Experimental evidence of a tripartite mutualism: bacteria protect ant fungus gardens from specialized parasites. *Oikos* 101, 91–102. https://doi.org/10.1034/j.1600-0706.2003.12036.x

Hall, T. A. (1999). BioEdit: a user-friendly biological sequence alignment editor and analysis program for Windows 95/98/NT. *Nucleic Acids Symp. Ser.* 41, 95–98

Haugland, R. L., and Heckman, J. L. (1998). Identification of putative sequence specific PCR primers for detection of the toxigenic fungal species *Stachybotrys chartarum*. *Mol. Cell. Probes* 12, 387–396. https://doi.org/10.1006/mcpr.1998.0197

Schoch, C. L., Seifert, K. A., Huhndorf, S., Robert, V., Spouge, J. L., Levesque, C. A., et al. (2012). Nuclear ribosomal internal transcribed spacer (ITS) region as a universal DNA barcode marker for Fungi. *Proc. Natl. Acad. Sci. U.S.A.* 109, 6241–6246. https://doi.org/10.1073/pnas.1117018109

Taerum, S. J., Cafaro, M. J., Little, A. E., Shultz, T. R., and Currie, C. R. (2007). Low host pathogen specificity in the leaf-cutting ant-microbe symbiosis. *Proc. R. Soc. B* 274, 1971–1978. https://doi.org/10.1098/rspb.2007.0431

White, T. J., Bruns, T., Lee, S. H., and Taylor, J. W. (1990). *PCR Protocols: a Guide to Methods and Application*. San Diego, CA: Academic Press, 315–322. doi:10.1016/B978-0-12- 372180-8.50042-1
